# Supplementary figures and images for: Effects of heat waves on cardiovascular and respiratory mortality in Rio de Janeiro, Brazil
Source: PLoS One. 2023 Mar 31;18(3):e0283899. doi: 10.1371/journal.pone.0283899 (PMC10065291; doi:10.1371/journal.pone.0283899)

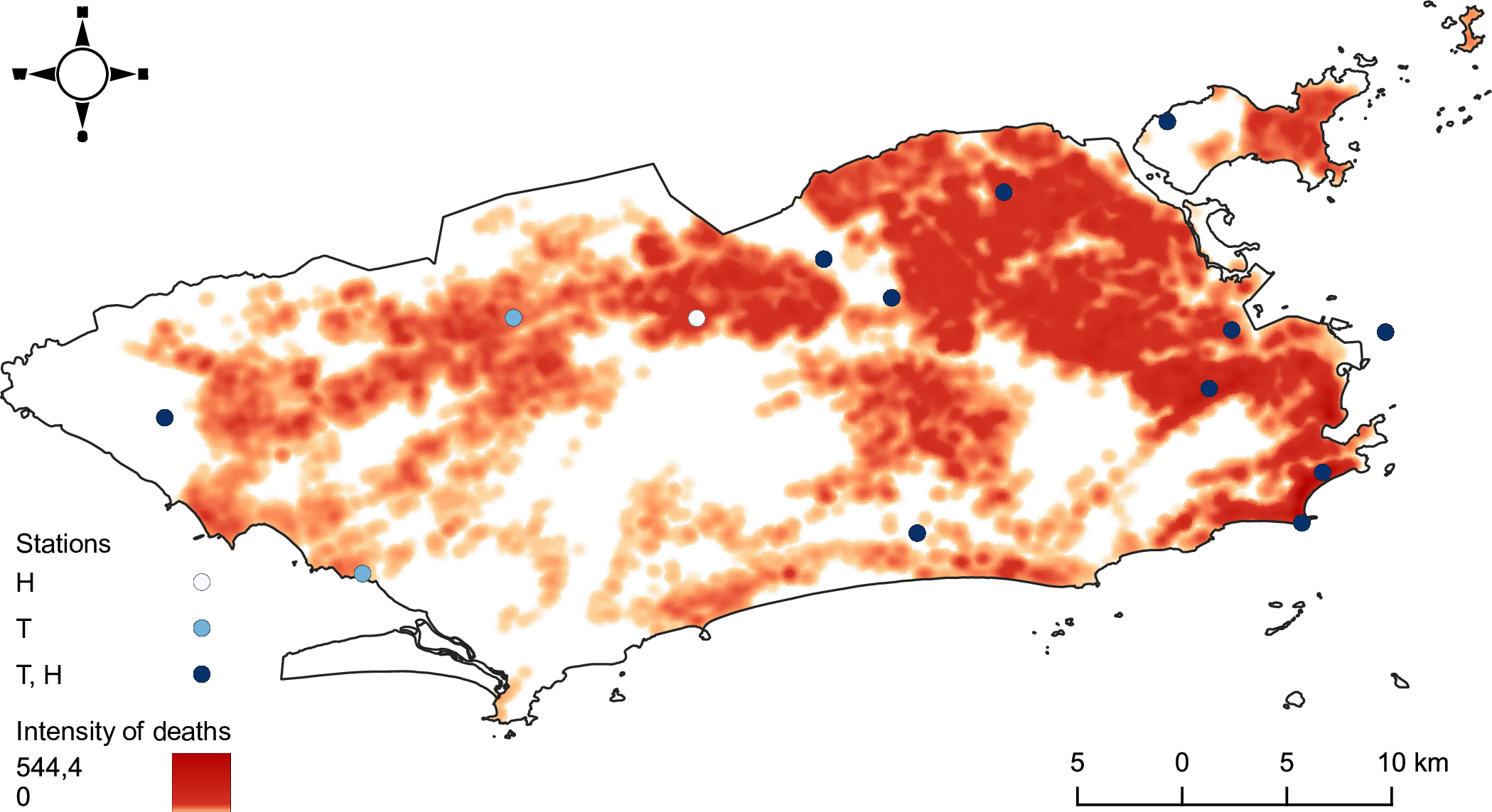

Supplement: S1 Fig — The heat map of deaths was created using a 500 m search radius. Source of the cartographic database: Brazilian Institute of Geography and Statistics. (TIF) [file pone.0283899.s001.tif]

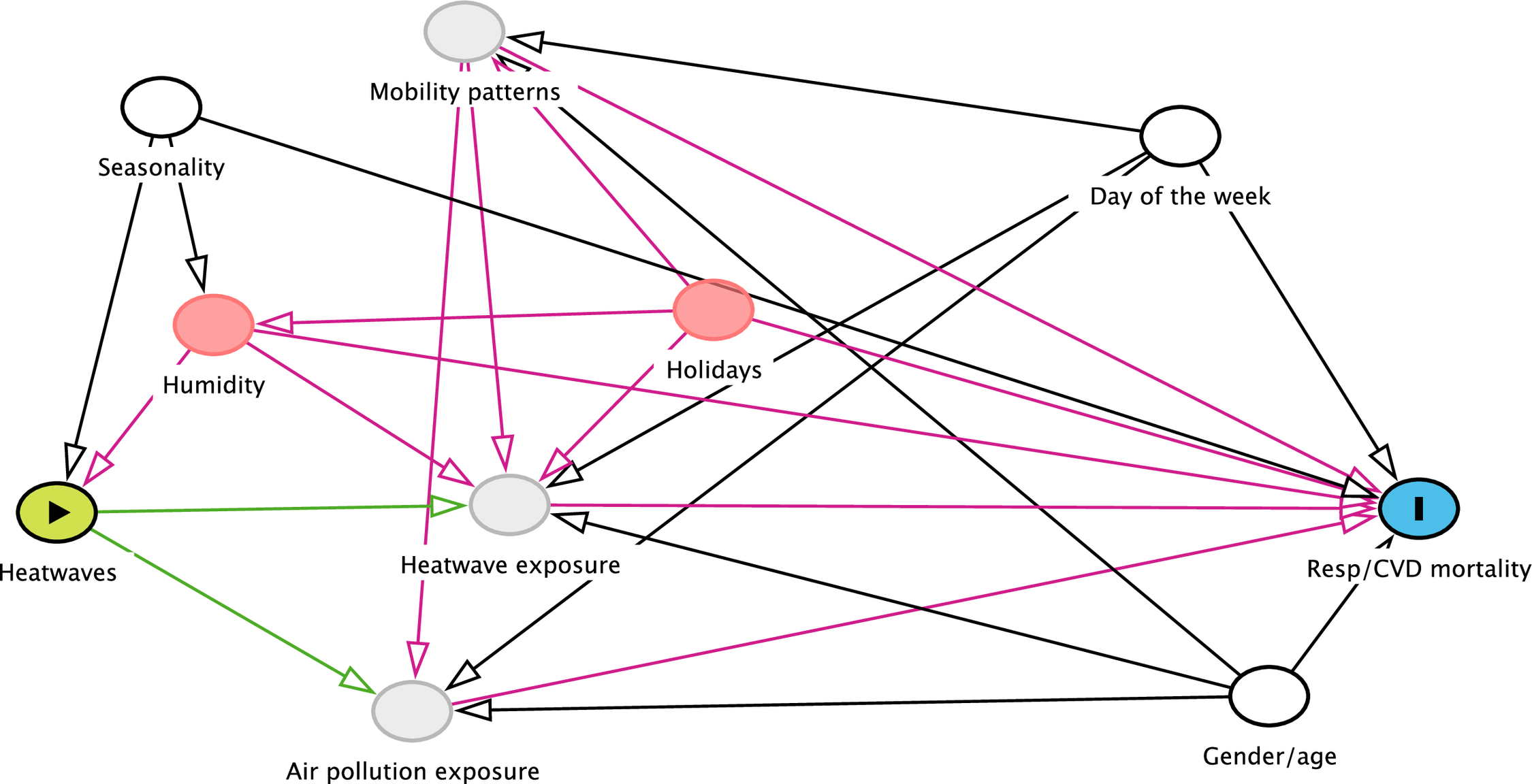

Supplement: S2 Fig — Causal pathways between exposure and outcome are represented by green arrows; noncausal pathways (back-door paths) by red arrows; unmeasured factors by grey nodes; confounders by red nodes; and factors controlled by design by white nodes. The sufficient adjustment set of variables to estimate the total effect of heat waves on mortality included humidity and public holidays. (TIF) [file pone.0283899.s002.tif]
