# Supplementary material for: Effects of heat waves on cardiovascular and respiratory mortality in Rio de Janeiro, Brazil
Source: PLoS One. 2023 Mar 31;18(3):e0283899. doi: 10.1371/journal.pone.0283899 (PMC10065291; doi:10.1371/journal.pone.0283899)
Supplement: S3 Fig — We used a natural cubic spline with 4 degrees of freedom for the lag effect. Effect estimates are reported as odds ratios (OR), and the dashes represent 95% confidence intervals. (PDF) [file pone.0283899.s003.pdf]

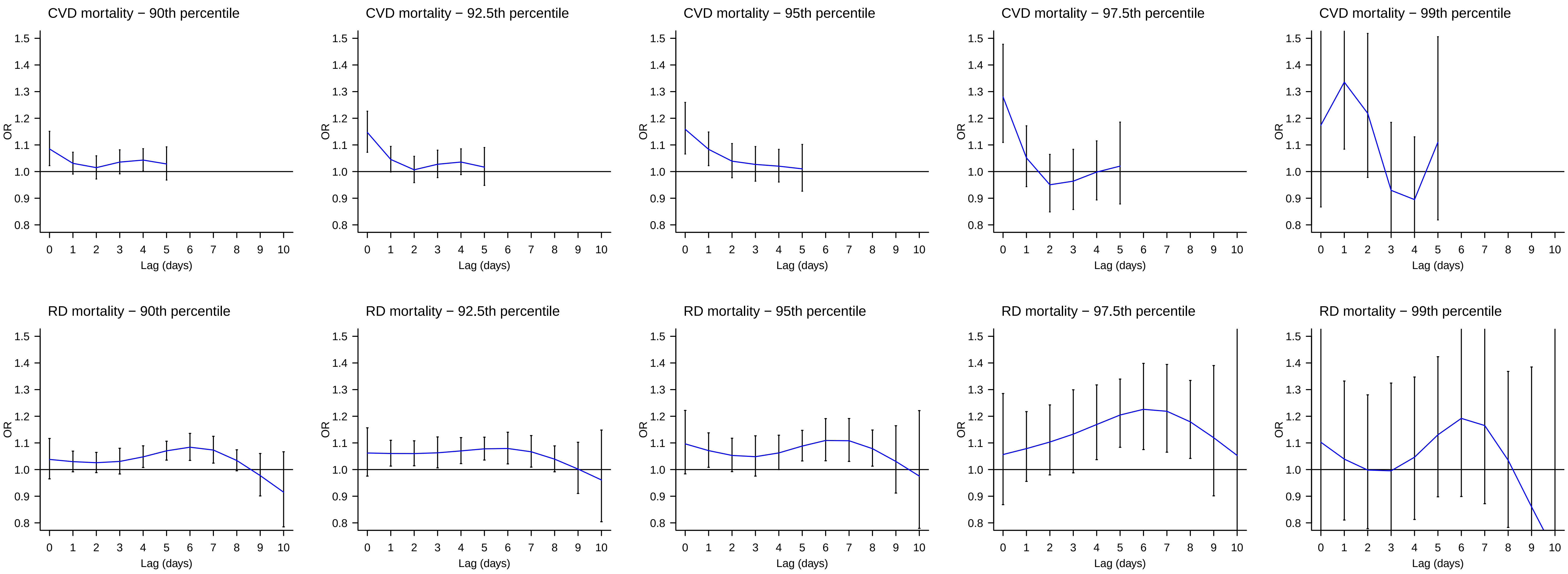
CVD mortality − 90th percentile
CVD mortality − 92.5th percentile
CVD mortality − 95th percentile
CVD mortality − 97.5th percentile
CVD mortality − 99th percentile
RD mortality − 90th percentile
RD mortality − 92.5th percentile
RD mortality − 95th percentile
RD mortality − 97.5th percentile
RD mortality − 99th percentile
OR
Lag (days)
