# Supplementary material for: Effects of heat waves on cardiovascular and respiratory mortality in Rio de Janeiro, Brazil
Source: PLoS One. 2023 Mar 31;18(3):e0283899. doi: 10.1371/journal.pone.0283899 (PMC10065291; doi:10.1371/journal.pone.0283899)
Supplement: S1 Table — (DOCX) [file pone.0283899.s004.docx]

### S1 Table. Percentage of missing data imputed in the daily series of meteorological variables for each monitor.

| Source | Frequency* | Station ID | Temperature | Relative humidity |
| --- | --- | --- | --- | --- |
| DCEA | Daily | 83054 | 9.1 | 9.1 |
| DCEA | Daily | 83115 | 8.9 | 8.9 |
| DCEA | Daily | 83746 | 1.7 | 1.7 |
| DCEA | Daily | 83748 | 11.4 | 10.8 |
| DCEA | Daily | 83755 | 6.0 | 6.1 |
| DCEA | Daily | 83743 | excluded | excluded |
| INMET | Daily | A621 | 1.6 | 1.7 |
| INMET | Daily | A652 | 0 | 0 |
| SMAC | Hourly | AV | 1.2 | 1.2 |
| SMAC | Hourly | BG | excluded | 2.6 |
| SMAC | Hourly | CA | excluded | excluded |
| SMAC | Hourly | CG | 1.5 | excluded |
| SMAC | Hourly | IR | 1.3 | 1.9 |
| SMAC | Hourly | PG | 2.1 | excluded |
| SMAC | Hourly | SC | 1.4 | 2.1 |
| SMAC | Hourly | SP | 2.3 | 2.0 |

* Record frequency of crude data provided.

DCEA: Department of Airspace Control (*Departamento de Controle do Espaço Aéreo*). INMET: National Institute of Meteorology (*Instituto Nacional de Meteorologia*). SMAC: Rio de Janeiro Municipal Secretariat for the Environment (*Secretaria Municipal de Meio Ambiente do Rio de Janeiro*). Stations with more than 20% missing data per year, and more than 15% over the study period, were excluded.

Additional information about data availability:

- Data from DCEA were obtained from the PROTIM/CPTEC/INPE (Portal de Tecnologia da Informação para Meteorologia, Centro de Previsão do Tempo e Estudos Climáticos, Instituto Nacional de Pesquisas Espaciais) system, website currently not working. Nowadays, the dataset from DCEA can be downloaded at: <https://bndmet.decea.mil.br/>.
- Data from INMET can be obtained from the INMET meteorological database system (BDMEP) at [https://bdmep.inmet.gov.br/#](https://bdmep.inmet.gov.br/);
- Data from SMAC can be obtained from the website DATA.RIO (<https://www.data.rio/maps/PCRJ::qualidade-do-ar-dados-hor%C3%A1rios/about>).
