# Supplementary material for: Effects of heat waves on cardiovascular and respiratory mortality in Rio de Janeiro, Brazil
Source: PLoS One. 2023 Mar 31;18(3):e0283899. doi: 10.1371/journal.pone.0283899 (PMC10065291; doi:10.1371/journal.pone.0283899)
Supplement: S2 Table — (DOCX) [file pone.0283899.s005.docx]

### S2 Table. Root mean square error (RMSE) and mean absolute error (MAE) values of cross-validation for the different sets of inverse distance weighting parameters.

| **Search distance** | **Temperature** | | **Relative humidity** | |
| --- | --- | --- | --- | --- |
|  | **RMSE** | **MAE** | **RMSE** | **MAE** |
| 10km | 2.027 | 1.712 | 6.120 | 5.200 |
| 15km | 1.854 | 1.560 | 6.024 | 5.026 |
| 20km | 1.872 | 1.552 | 5.826 | 5.826 |
| >20km | 1.720 | 1.412 | 5.811 | 4.801 |
