# Supplementary material for: Effects of heat waves on cardiovascular and respiratory mortality in Rio de Janeiro, Brazil
Source: PLoS One. 2023 Mar 31;18(3):e0283899. doi: 10.1371/journal.pone.0283899 (PMC10065291; doi:10.1371/journal.pone.0283899)
Supplement: S3 Table — For all definitions, we used a duration of 2 days or more. (DOCX) [file pone.0283899.s006.docx]

### S3 Table. Mean threshold values for each heat wave definition. For all definitions, we used a duration of 2 days or more.

| **Abbreviation** | **Percentile** | **Temperature value (ºC)** |
| --- | --- | --- |
| HW_90 | 90^th^ | 31.03 |
| HW_925 | 92.5^th^ | 31.30 |
| HW_95 | 95^th^ | 31.68 |
| HW_975 | 97.5^th^ | 32.19 |
| HW_99 | 99^th^ | 32.71 |
