# Supplementary material for: Effects of heat waves on cardiovascular and respiratory mortality in Rio de Janeiro, Brazil
Source: PLoS One. 2023 Mar 31;18(3):e0283899. doi: 10.1371/journal.pone.0283899 (PMC10065291; doi:10.1371/journal.pone.0283899)
Supplement: S4 Table — (DOCX) [file pone.0283899.s007.docx]

### S4 Table. Results of sensitivity analysis

|  | Cardiovascular mortality | | | | | Respiratory mortality | | | | |
| --- | --- | --- | --- | --- | --- | --- | --- | --- | --- | --- |
|  | HW90 | HW925 | HW95 | HW975 | HW99 | HW90 | HW925 | HW95 | HW975 | HW99 |
| main | 1.26  (1.14-1.40) | 1.31  (1.15-1.49) | 1.38  (1.15-1.66) | 1.26  (0.86-1.83) | 1.76  (0.80-3.88) | 1.36  (1.03-1.81) | 1.69  (1.19-2.40) | 2.00  (1.23-3.25) | 4.17  (1.55-11.18) | 1.15  (0.15-8.90) |
| bs for lag | 1.25  (1.13-1.39) | 1.29  (1.13-1.47) | 1.37  (1.14-1.65) | 1.26  (0.86-1.83) | 1.74  (0.79-3.84) | 1.23  (0.86-1.75) | 1.66  (1.08-2.54) | 2.07  (1.18-3.63) | 4.84  (1.65-14.18) | 1.66  (0.18-15.57) |
| ns_3df | 1.26  (1.14-1.40) | 1.31  (1.15-1.49) | 1.38  (1.15-1.66) | 1.25  (0.86-1.83) | 1.84  (0.84-4.04) | 1.48  (1.15-1.90) | 1.75  (1.27-2.41) | 2.18  (1.39-3.42) | 4.44  (1.74-11.3) | 1.54  (0.22-10.64) |
| ns_5df | 1.25  (1.13-1.39) | 1.29  (1.13-1.47) | 1.37  (1.14-1.65) | 1.26  (0.86-1.83) | 1.76  (0.80-3.86) | 1.31  (0.96-1.79) | 1.72  (1.17-2.51) | 2.10  (1.26-3.5) | 4.59  (1.67-12.64) | 1.50  (0.18-12.3) |
| 3-day lag | 1.20  (1.10-1.32) | 1.24  (1.11-1.39) | 1.34  (1.16-1.56) | 1.23  (0.91-1.67) | 1.95  (1.05-3.60) | 1.17  (1.02-1.33) | 1.25  (1.06-1.47) | 1.20  (0.96-1.49) | 1.08  (0.69-1.68) | 0.99  (0.39-2.56) |
| 10 or 5-day lag | 1.17  (0.96-1.42) | 1.23  (0.96-1.56) | 1.48  (1.05-2.07) | 1.13  (0.58-2.20) | 0.74  (0.19-2.98) | 1.3  (1.11-1.51) | 1.49  (1.24-1.80) | 1.48  (1.14-1.92) | 1.65  (0.96-2.86) | 1.22  (0.38-3.91) |
| without humidity | 1.32  (1.20-1.46) | 1.38  (1.22-1.56) | 1.48  (1.24-1.77) | 1.39  (0.96-2.02) | 2.08  (0.95-4.55) | 1.45  (1.11-1.91) | 1.82  (1.29-2.56) | 2.21  (1.38-3.56) | 4.90  (1.85-13.00) | 1.49  (0.20-11.42) |
| with temp | 1.25  (1.08-1.44) | 1.30  (1.09-1.56) | 1.36  (1.07-1.72) | 1.05  (0.68-1.63) | 1.54  (0.66-3.62) | 1.03  (0.71-1.49) | 1.29  (0.82-2.04) | 1.30  (0.70-2.41) | 2.16  (0.69-6.72) | 0.27  (0.03-2.38) |

^bs: quadratic b-spline. df: degrees of freedom. ns: natural cubic spline.^
